# Supplementary material for: Gli1 marks a sentinel muscle stem cell population for muscle regeneration
Source: Nat Commun. 2023 Nov 1;14:6993. doi: 10.1038/s41467-023-42837-8 (PMC10620419; doi:10.1038/s41467-023-42837-8)
Supplement: Supplementary file 1 — Supplementary Information [file 41467_2023_42837_MOESM1_ESM.pdf]

Supplementary Materials for

Gli1 marks a sentinel muscle stem cell population for muscle regeneration

Jiayin Peng, Lili Han, Biao Liu, Jiawen Song, Yuang Wang, Kunpeng Wang, Qian Guo, XinYan Liu, Yu Li, Jujin Zhang, Wenqing Wu, Sheng Li, Xin Fu, Cheng-le Zhuang, Weikang Zhang, Shengbao Suo, Ping Hu#, Yun Zhao#

Correspondence to: yunzhao@sibcb.ac.cn (Y.Z.), hup@sibcb.ac.cn (P.H.)

This PDF file includes:

Figs. S1 to S10

Tables S1 to S3

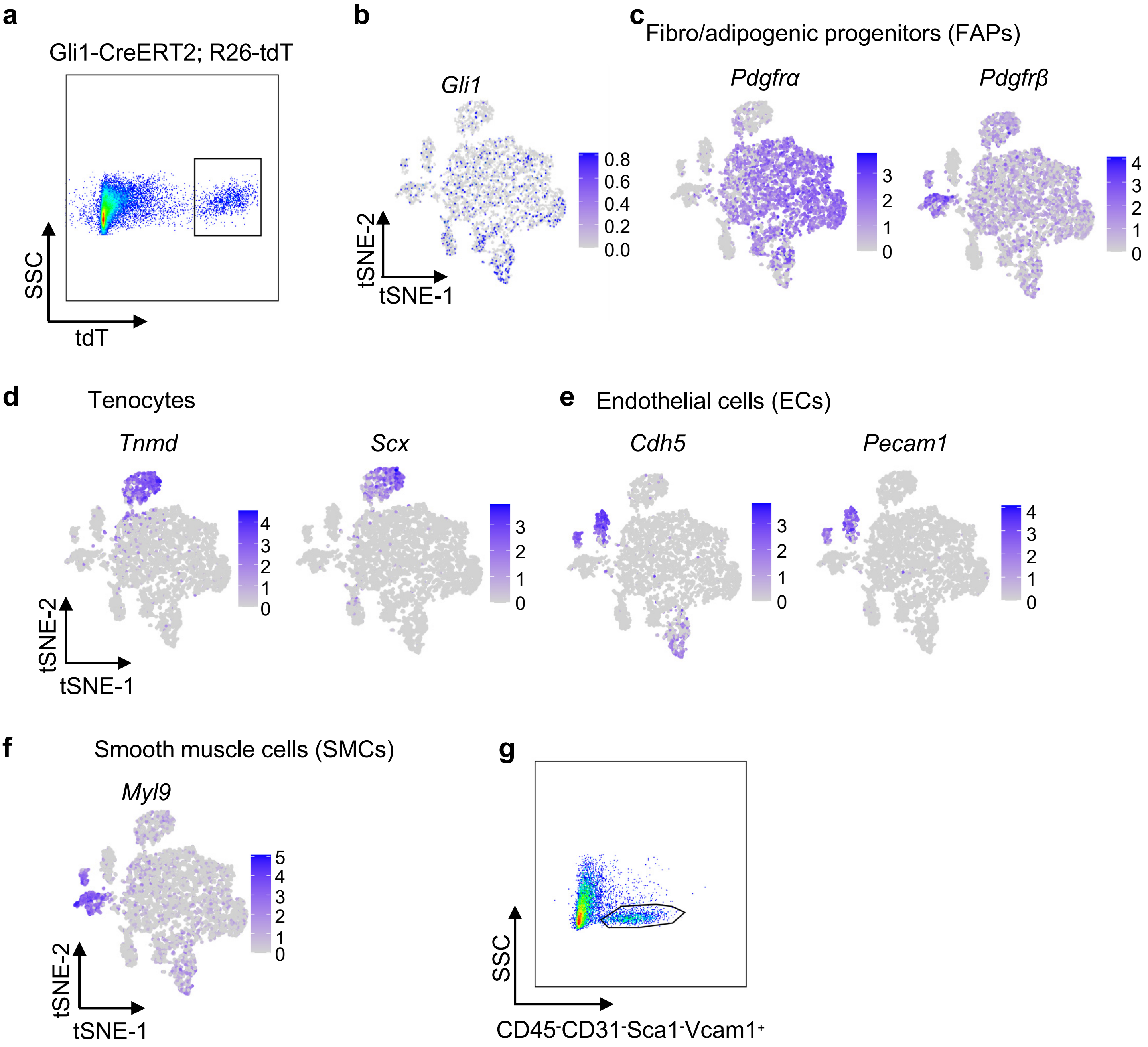
a
Gli1-CreERT2; R26-tdT
SSC
tdT
b
Gli1
tSNE-2
tSNE-1
0.8
0.6
0.4
0.2
0.0
c
Fibro/adipogenic progenitors (FAPs)
Pdgfrα
Pdgfrβ
d
Tenocytes
Tnmd
Scx
e
Endothelial cells (ECs)
Cdh5
Pecam1
f
Smooth muscle cells (SMCs)
Myl9
g
SSC
CD45-CD31-Sca1-Vcam1+

Supplementary Fig. 1 scRNA-seq analysis of Gli1$^+$ cell distribution.

a Representative profile of FACS-sorted tdT$^+$ (Gli1$^+$) cell populations from Gli1-CreERT2; R26-tdT mice for scRNA-seq (n = 3). Box represented the tdT$^+$ (Gli1$^+$) cell populations. **b** Distribution of gene for *Gli1* across all subpopulations. **c** Distribution of genes for fibro/adipogenic progenitors (FAPs) (*Pdgfrα* and *Pdgfrβ*) across all subpopulations. **d** Distribution of genes for tenocytes (*Tnmd* and *Scx*) across all subpopulations. **e** Distribution of genes for endothelial cells (ECs) (*Cdh5* and *Pecam1*) across all subpopulations. **f** Distribution of gene for smooth muscle cells (SMCs) (*Myl9*) across all subpopulations. **g** Representative profiles of FACS-sorted MuSCs from C57 WT mice for scRNA-seq (n = 3). Box represented the MuSC populations.

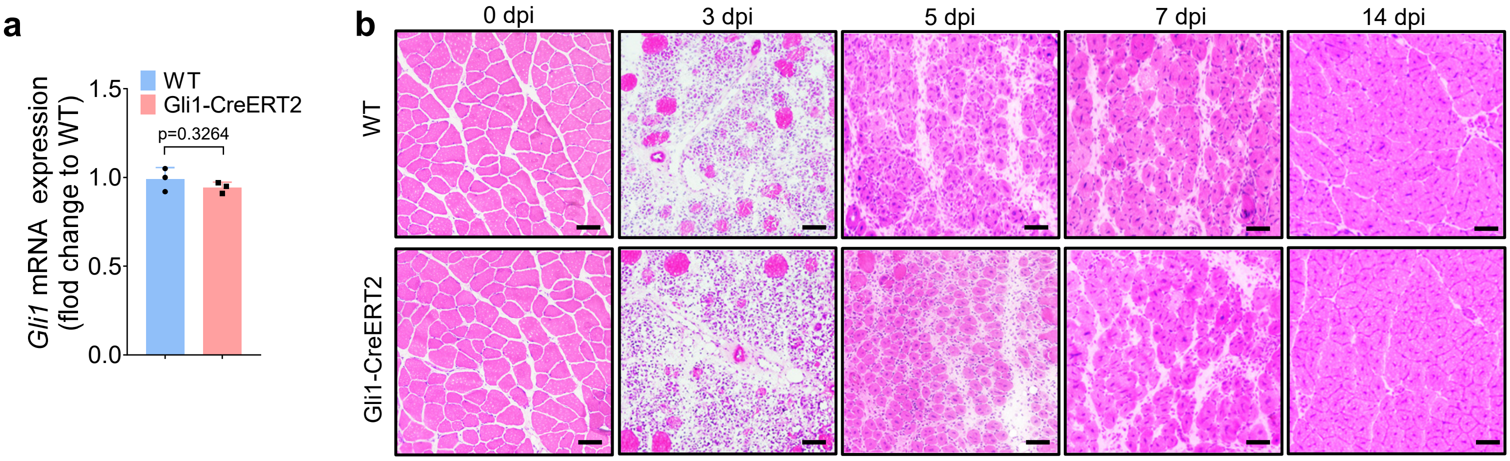

a
Gli1 mRNA expression (flod change to WT)
1.5
1.0
0.5
0.0
WT
Gli1-CreERT2
p=0.3264
b
0 dpi
3 dpi
5 dpi
7 dpi
14 dpi
WT
Gli1-CreERT2

Supplementary Fig. 2 Gli1-CreERT2 heterozygous does not affect muscle regeneration.

a RT-qPCR for *Gli1* in MuSCs from Gli1-CreERT2 heterozygous compared to WT control mice (n = 3). **b** H&E-stained sections of injured TA muscles at different time point from WT control and Gli1-CreERT2 heterozygous mice (n = 3). Scale bars, 50 μm. Data are presented as mean ± SEM; Statistical significance was determined by two-tailed unpaired Student's *t* test (**a**). All numbers (n) are biologically independent experiments. Source data is provided in the Source Data File.

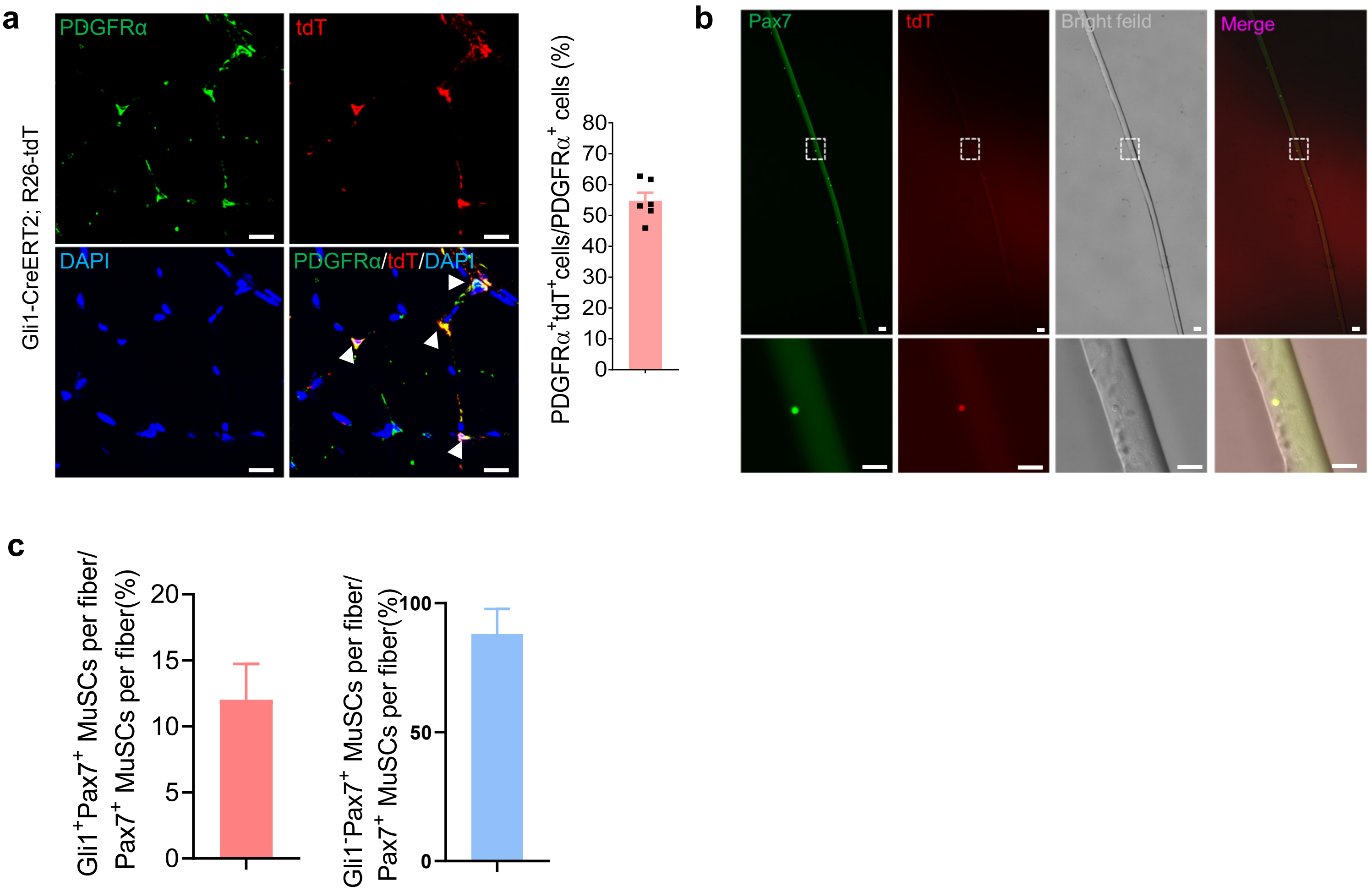

a
PDGFRα
tdT
DAPI
PDGFRα/tdT/DAPI
Gli1-CreERT2; R26-tdT
PDGFRα+tdT+cells/PDGFRα+ cells (%)
80
70
60
50
40
30
20
10
0
b
Pax7
tdT
Bright feild
Merge
c
Gli1+Pax7+ MuSCs per fiber/
Pax7+ MuSCs per fiber(%)
20
15
10
5
0
Gli1-Pax7+ MuSCs per fiber/
Pax7+ MuSCs per fiber(%)
100
50
0

Supplementary Fig. 3 Characterization of Gli1$^+$ cells in skeletal muscles and myofibers.

a Left panel: Immunofluorescence staining for tdT (red), PDGFRα (green) and DAPI (blue) on TA muscle sections from Gli1-CreERT2; R26-tdT mice (n = 6). Arrow heads indicated the tdT$^+$ (Gli1$^+$) PDGFRα$^+$ cells. Scale bars, 20 μm. Right panel: Quantification of the percentage of PDGFRα$^+$ cells expressing tdT (n = 6). **b** Immunofluorescence staining for tdT (red), Pax7 (green) and DAPI (blue) in isolated single EDL myofibers (n = 13) from Gli1-CreERT2; R26-tdT mice. The boxed regions are magnified in the panels below with split channels. Scale bars, 20 μm. **c** Left panel: Quantification analysis of the per fiber of Pax7$^+$ MuSCs cells expressing tdT$^+$ (Gli1$^+$) (n = 13). Right panel: Quantification analysis of the per fiber of Pax7$^+$ MuSCs not expressing tdT$^-$ (Gli1$^-$) (n = 13). Data are presented as mean ± SEM. All numbers (n) are biologically independent experiments. Source data is provided in the Source Data File.

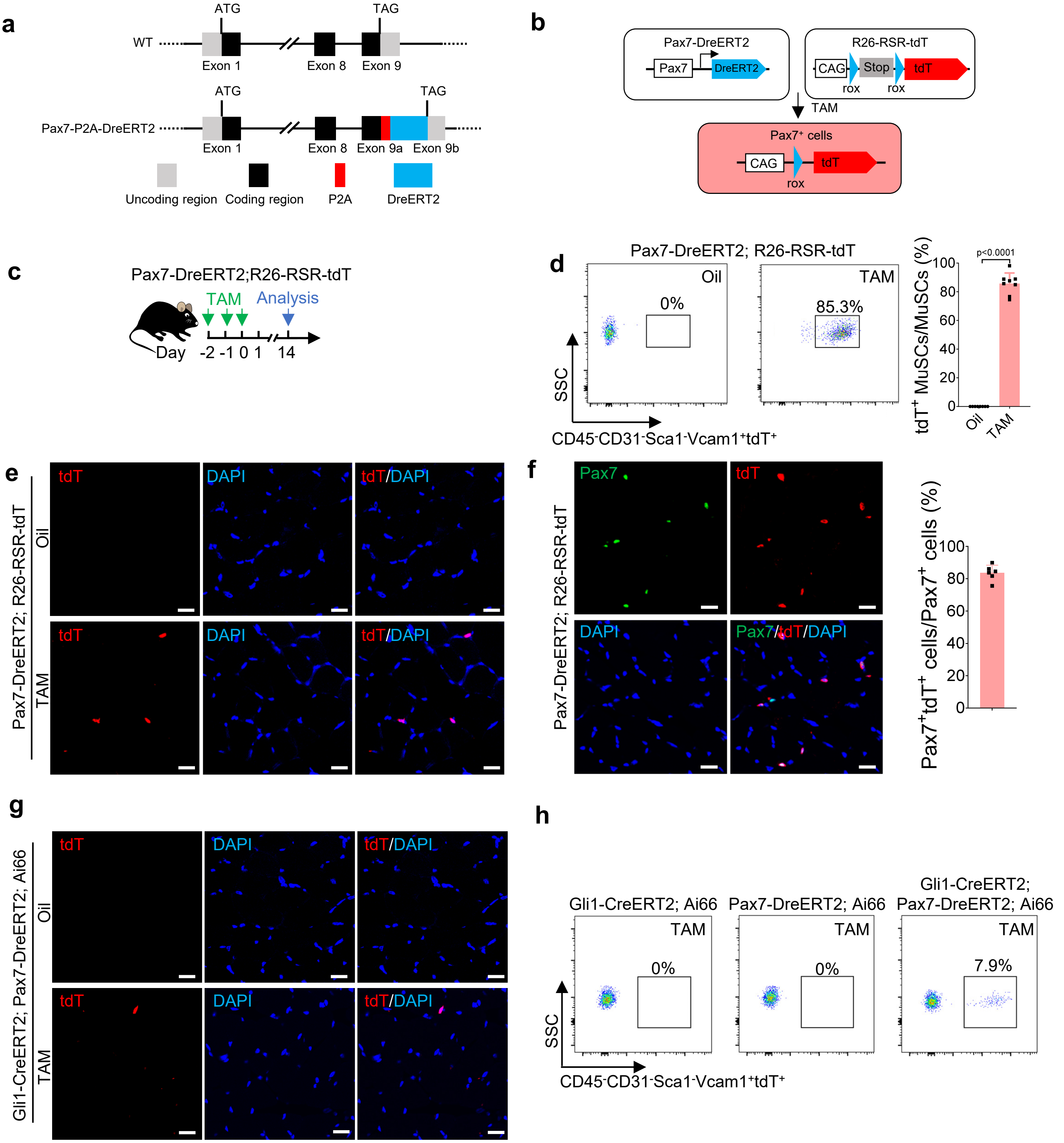
a
ATG
TAG
WT
Exon 1
Exon 8
Exon 9
ATG
TAG
Pax7-P2A-DreERT2
Exon 1
Exon 8
Exon 9a
Exon 9b
Uncoding region
Coding region
P2A
DreERT2
b
Pax7-DreERT2
Pax7
DreERT2
R26-RSR-tdT
CAG
Stop
tdT
rox
rox
TAM
Pax7⁺ cells
CAG
tdT
rox
c
Pax7-DreERT2;R26-RSR-tdT
TAM
Analysis
Day
-2 -1 0 1
14
d
Pax7-DreERT2; R26-RSR-tdT
Oil
0%
TAM
85.3%
SSC
CD45⁻CD31⁻Sca1⁻Vcam1⁺tdT⁺
tdT⁺ MuSCs/MuSCs (%)
p<0.0001
Oil
TAM
e
Pax7-DreERT2; R26-RSR-tdT
Oil
TAM
tdT
DAPI
tdT/DAPI
f
Pax7-DreERT2; R26-RSR-tdT
Pax7
tdT
DAPI
Pax7/tdT/DAPI
Pax7⁺tdT⁺ cells/Pax7⁺ cells (%)
g
Gli1-CreERT2; Pax7-DreERT2; Ai66
Oil
TAM
tdT
DAPI
tdT/DAPI
h
Gli1-CreERT2; Ai66
Pax7-DreERT2; Ai66
Gli1-CreERT2; Pax7-DreERT2; Ai66
TAM
0%
0%
7.9%
SSC
CD45⁻CD31⁻Sca1⁻Vcam1⁺tdT⁺

Supplementary Fig. 4 Generation and characterization of Pax7-DreERT2 mouse allele.

a Scheme of knock-in strategy for generation of Pax7-DreERT2 mouse. **b** Scheme of genetic lineage tracing strategy by Pax7-DreERT2; R26-RSR-tdT mice. **c** Scheme of the experimental strategy. **d** Left panel: Representative flow cytometry plots and the percentage of MuSCs expressing tdT with oil or TAM treatment (n = 8). Right panel: Quantification of the percentage of MuSCs expressing tdT (n = 8). **e** Immunofluorescence staining for tdT (red) and DAPI (blue) on TA muscle sections of Pax7-DreERT2; R26-RSR-tdT mice at day 14 with oil or TAM treatment (n = 6). Scale bars, 50 µm. **f** Left panel: Immunofluorescence staining for tdT (red), Pax7 (green) and DAPI (blue) on TA muscle sections from Pax7-DreERT2; R26-RSR-tdT mice (n = 6). Scale bars, 50 µm. Right panel: Quantification of the percentage of $Pax7^+$ cells expressing tdT (n = 6). **g** Immunofluorescence staining for tdT (red) and DAPI (blue) on TA muscle sections of Gli1-CreERT2; Pax7-DreERT2; Ai66 mice at day 14 with oil or TAM treatment (n = 5). Scale bars, 50 µm. **h** Representative flow cytometry plots of MuSCs expressing tdT (n = 5). The percentage of tdT^+ ($Gli1^+$ $Pax7^+$) MuSCs was 7.9%. Data are presented as mean ± SEM; Statistical significance was determined by two-tailed unpaired Student's *t* test (**d**). All numbers (n) are biologically independent experiments. Source data is provided in the Source Data File.

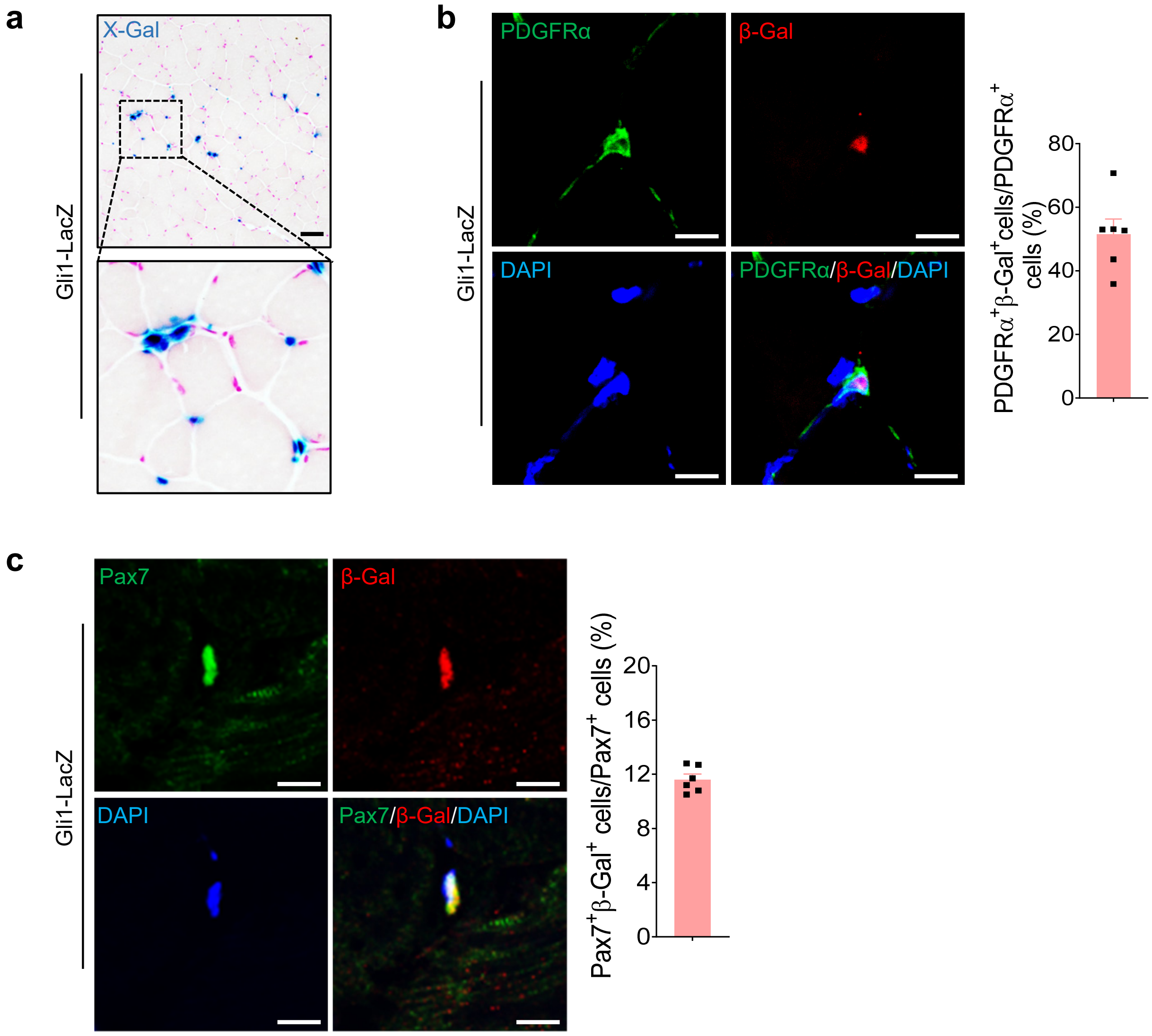
a
X-Gal
Gli1-LacZ
b
PDGFRα
β-Gal
DAPI
PDGFRα/β-Gal/DAPI
Gli1-LacZ
PDGFRα+β-Gal+cells/PDGFRα+ cells (%)
0
20
40
60
80
c
Pax7
β-Gal
DAPI
Pax7/β-Gal/DAPI
Gli1-LacZ
Pax7+β-Gal+ cells/Pax7+ cells (%)
0
4
8
12
16
20

Supplementary Fig. 5 Gli1 is expressed in MuSCs.

a Representative histological images of X-Gal-stained TA muscle sections from Gli1-LacZ mice (n = 6). Scale bars, 200 μm. **b** Left panel: Immunofluorescence staining for β-Gal (red), PDGFRα (green) and DAPI (blue) on TA muscle sections (n = 6). Scale bars, 10 μm. Right panel: Quantification of the percentage of PDGFRα$^+$ cells expressing β-Gal (n = 6). **c** Left panel: Immunofluorescence staining for β-Gal (red), Pax7 (green) and DAPI (blue) on TA muscle sections from Gli1-LacZ mice (n = 6). Scale bars, 10 μm. Right panel: Quantification of the percentage of Pax7$^+$ cells expressing β-Gal. The percentage of β-Gal$^+$ (Gli1$^+$) Pax7$^+$ cells was ~11.6% (n = 6). Data are presented as mean ± SEM. All numbers (n) are biologically independent experiments. Source data is provided in the Source Data File.

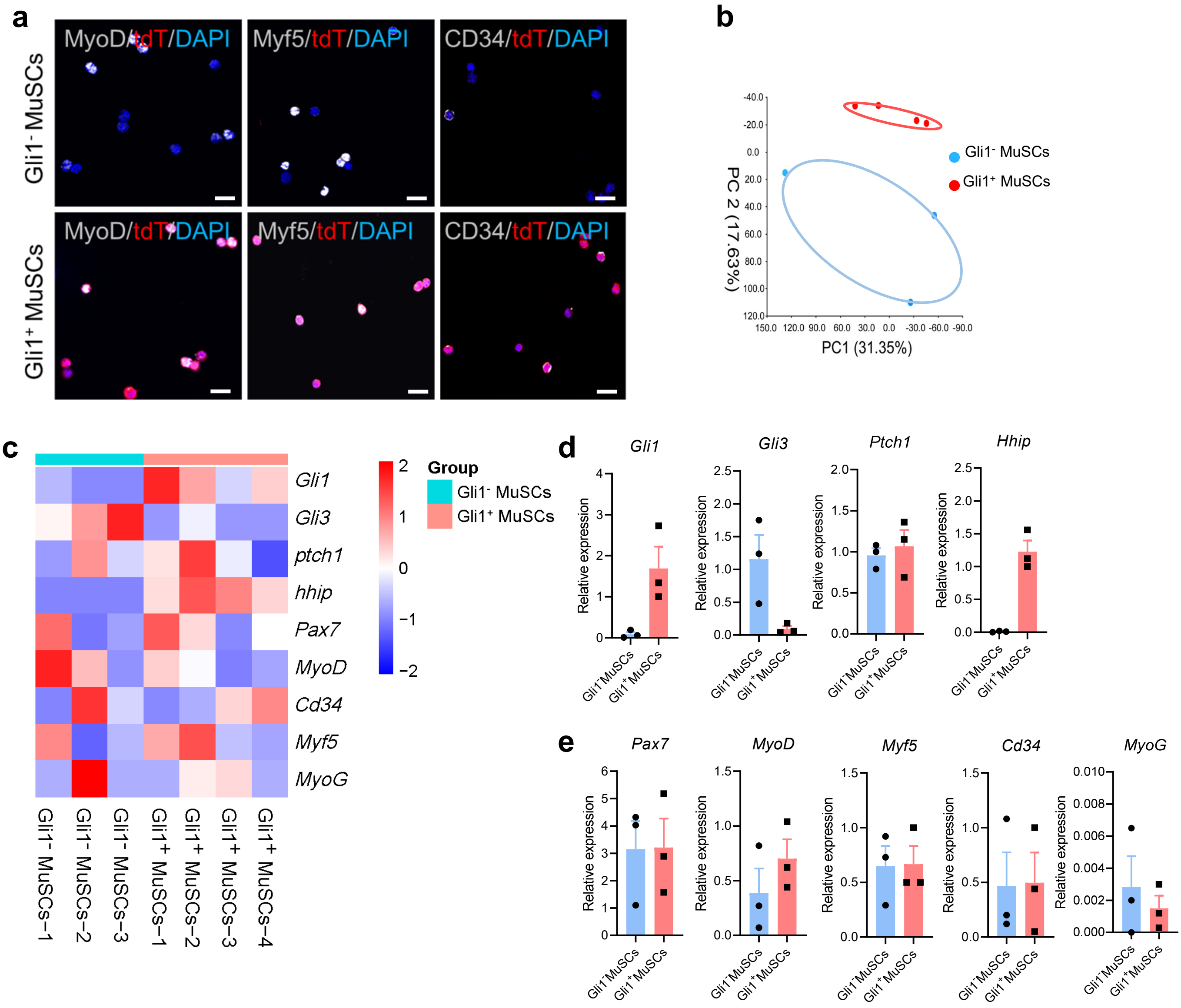
a
MyoD/tdT/DAPI
Myf5/tdT/DAPI
CD34/tdT/DAPI
Gli1- MuSCs
Gli1+ MuSCs
b
PC 2 (17.63%)
PC1 (31.35%)
Gli1- MuSCs
Gli1+ MuSCs
c
Group
Gli1- MuSCs
Gli1+ MuSCs
Gli1
Gli3
ptch1
hhip
Pax7
MyoD
Cd34
Myf5
MyoG
Gli1- MuSCs-1
Gli1- MuSCs-2
Gli1- MuSCs-3
Gli1+ MuSCs-1
Gli1+ MuSCs-2
Gli1+ MuSCs-3
Gli1+ MuSCs-4
d
Gli1
Gli3
Ptch1
Hhip
Relative expression
e
Pax7
MyoD
Myf5
Cd34
MyoG
Relative expression

Supplementary Fig. 6 The expression levels of the Hh signal pathway and myogenic genes in Gli1^{-} MuSCs and Gli1^{+} MuSCs.

a Immunofluorescence staining for MyoD/Myf5/CD34 (white), tdT (red) and DAPI (blue) on tdT^{-} (Gli1^{-}) and tdT^{+} (Gli1^{+}) MuSCs (n = 3). Scale bars, 20 μm. **b** Principal component analysis (PCA) of the transcriptome data. **c** Heatmap of of the Hh signal pathway genes and myogenic genes. n = 3 mice for tdT^{-} (Gli1^{-}) MuSCs, n = 4 mice for tdT^{+} (Gli1^{+}) MuSCs. **d, e** Relative mRNA levels of the Hh signal pathway genes (**d**) and myogenic genes (**e**) were measured by RT-qPCR (n = 3). The red histogram represents gene expression level in tdT^{+} (Gli1^{+}) MuSCs. The blue histogram represents gene expression level in tdT^{-} (Gli1^{-}) MuSCs. Data are presented as mean ± SEM. All numbers (n) are biologically independent experiments. Source data is provided in the Source Data File.

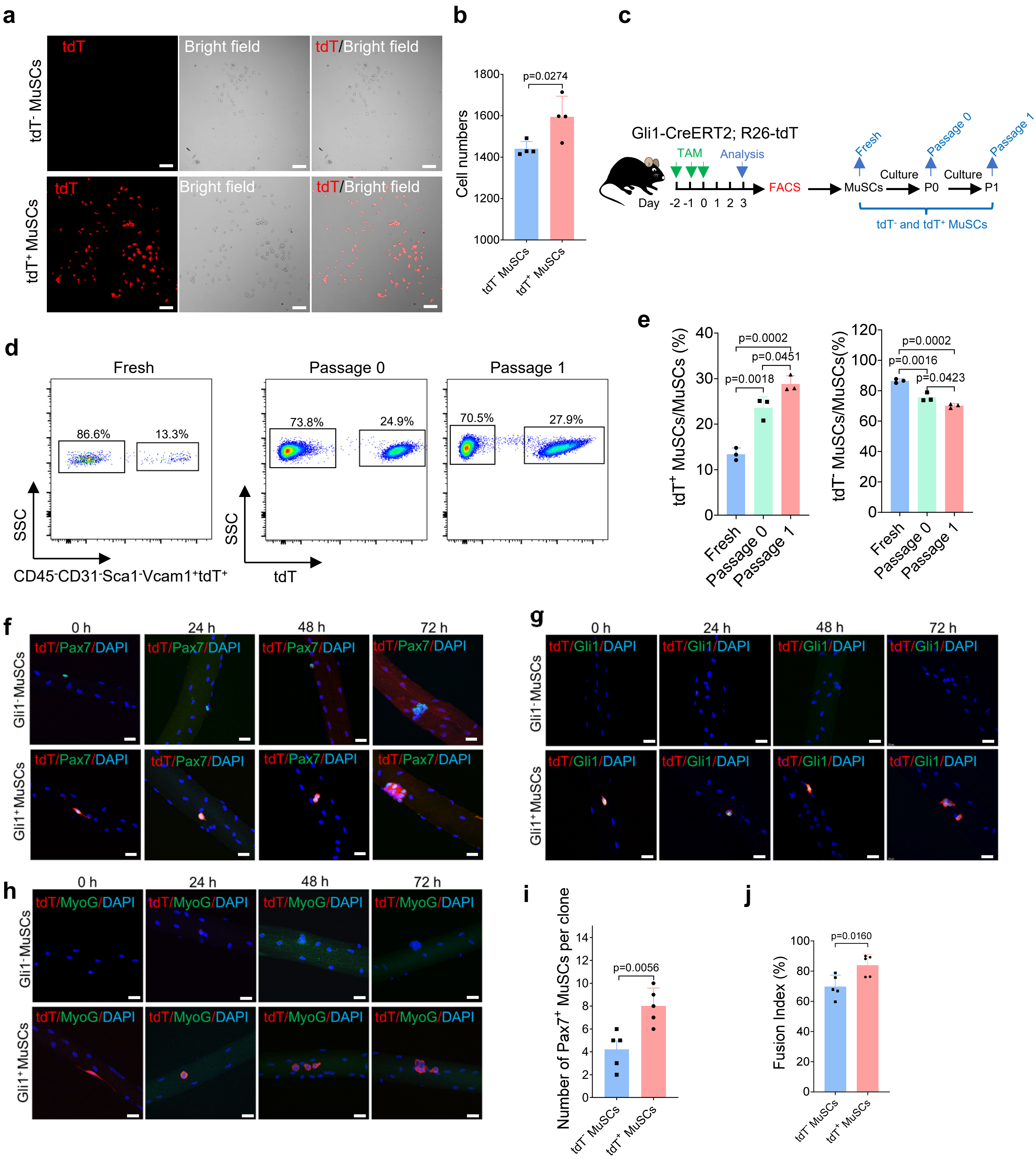
a
tdT- MuSCs
tdT+ MuSCs
tdT
Bright field
tdT/Bright field
b
Cell numbers
p=0.0274
tdT- MuSCs
tdT+ MuSCs
c
Gli1-CreERT2; R26-tdT
TAM
Analysis
Day -2 -1 0 1 2 3
FACS
MuSCs
Culture
P0
P1
Fresh
Passage 0
Passage 1
tdT- and tdT+ MuSCs
d
Fresh
Passage 0
Passage 1
86.6%
13.3%
73.8%
24.9%
70.5%
27.9%
SSC
CD45-CD31-Sca1-Vcam1+tdT+
tdT
e
tdT+ MuSCs/MuSCs (%)
tdT- MuSCs/MuSCs(%)
p=0.0002
p=0.0451
p=0.0018
p=0.0016
p=0.0423
Fresh
Passage 0
Passage 1
f
0 h
24 h
48 h
72 h
tdT/Pax7/DAPI
Gli1-MuSCs
Gli1+MuSCs
g
tdT/Gli1/DAPI
h
tdT/MyoG/DAPI
i
Number of Pax7+ MuSCs per clone
p=0.0056
j
Fusion Index (%)
p=0.0160

Supplementary Fig. 7 Gli1⁺ MuSCs undergo distinct numbers changes *in vitro*.

a, **b** Equal numbers (1,000 cells) of FACS-isolated tdT^+ ($Gli1^+$) or tdT^- ($Gli1^-$) MuSCs were cultured for 96 h (**a**, tdT: red) and quantification of the numbers of cells (**b**) (n = 4). Scale bars, 50 μm. **c** Schematic showing the experimental strategy. **d** FACS-sorted MuSCs were continuously passaged, and cell count determined at every passage is plotted. Flow cytometry analysis of the percentage of MuSCs expressing tdT (n = 3). **e** Quantifications of the percentage of tdT^+ ($Gli1^+$) (Left panel) or tdT^- ($Gli1^-$) (Right panel) MuSCs (n = 3). **f** Immunofluorescence staining for tdT (red), Pax7 (green), and DAPI (blue) on the single EDL myofibers isolated from Gli1-CreERT2; R26-tdT mice and cultured for 0 h, 24 h, 48 h and 72 h (n = 5). Scale bars, 20 μm. **g** Immunofluorescence staining for tdT (red), Gli1 (green), and DAPI (blue) on the single EDL myofibers isolated from Gli1-CreERT2; R26-tdT mice and cultured for 0 h, 24 h, 48 h and 72 h (n = 5). Scale bars, 20 μm. **h** Immunofluorescence staining for tdT (red), MyoG (green), and DAPI (blue) on the single EDL myofibers isolated from Gli1-CreERT2; R26-tdT mice and cultured for 0 h, 24 h, 48 h and 72 h (n = 5). Scale bars, 20 μm. **i** Quantifications of the number of $Pax7^+tdT^+$ ($Gli1^+$) or $Pax7^+tdT^-$ ($Gli1^-$) MuSCs per clone on muscle fibers (n = 5). **j** Fusion index of $MyHC^+$ myotubes differentiated from tdT^+ ($Gli1^+$) or tdT^- ($Gli1^-$) MuSCs. Cells were collected 2 days post differentiation (n = 5). Data are presented as mean ± SEM; Statistical significance was determined by two-tailed unpaired Student's *t* test (**b, i, j**) or one-way ANOVA (**e**). All numbers (n) are biologically independent experiments. Source data is provided in the Source Data File.

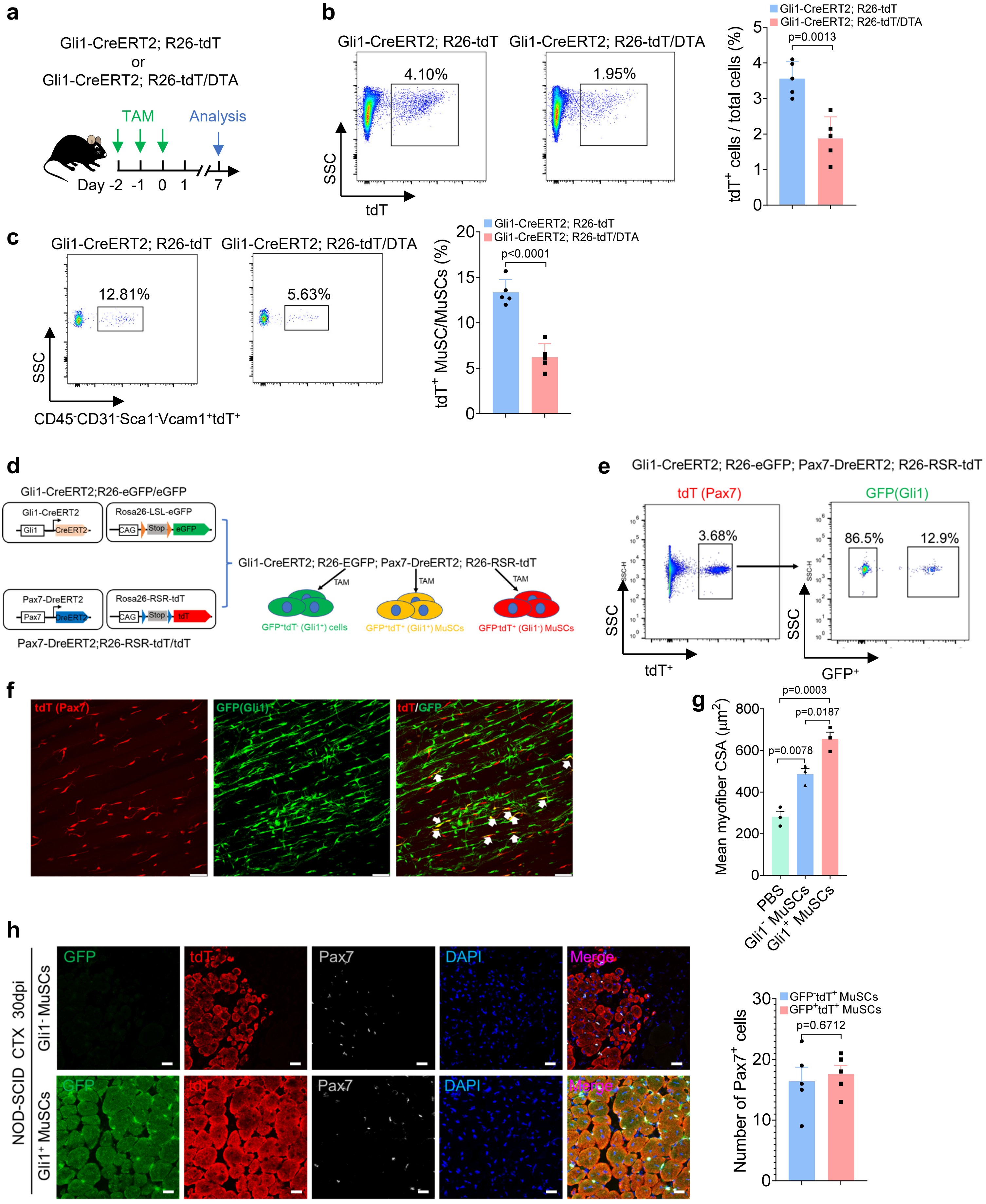
a
Gli1-CreERT2; R26-tdT
or
Gli1-CreERT2; R26-tdT/DTA
TAM
Analysis
Day -2 -1 0 1 7
b
Gli1-CreERT2; R26-tdT
Gli1-CreERT2; R26-tdT/DTA
4.10%
1.95%
SSC
tdT
Gli1-CreERT2; R26-tdT
Gli1-CreERT2; R26-tdT/DTA
p=0.0013
tdT+ cells / total cells (%)
c
Gli1-CreERT2; R26-tdT
Gli1-CreERT2; R26-tdT/DTA
12.81%
5.63%
SSC
CD45-CD31-Sca1-Vcam1+tdT+
Gli1-CreERT2; R26-tdT
Gli1-CreERT2; R26-tdT/DTA
p<0.0001
tdT+ MuSC/MuSCs (%)
d
Gli1-CreERT2;R26-eGFP/eGFP
Gli1-CreERT2
Rosa26-LSL-eGFP
Pax7-DreERT2
Rosa26-RSR-tdT
Pax7-DreERT2;R26-RSR-tdT/tdT
Gli1-CreERT2; R26-EGFP; Pax7-DreERT2; R26-RSR-tdT
TAM
GFP+tdT- (Gli1+) cells
GFP+tdT+ (Gli1+) MuSCs
GFP-tdT+ (Gli1-) MuSCs
e
Gli1-CreERT2; R26-eGFP; Pax7-DreERT2; R26-RSR-tdT
tdT (Pax7)
GFP(Gli1)
3.68%
86.5%
12.9%
SSC
tdT+
GFP+
f
tdT (Pax7)
GFP(Gli1)
tdT/GFP
g
p=0.0003
p=0.0187
p=0.0078
Mean myofiber CSA (μm2)
PBS
Gli1- MuSCs
Gli1+ MuSCs
h
NOD-SCID CTX 30dpi
Gli1- MuSCs
Gli1+ MuSCs
GFP
tdT
Pax7
DAPI
Merge
GFP-tdT+ MuSCs
GFP+tdT+ MuSCs
p=0.6712
Number of Pax7+ cells

Supplementary Fig. 8 Gli1$^+$ MuSCs are important for muscle regeneration *in vivo*.

a Scheme of the experimental strategy. **b** Left panel: Fluorescence-activated cell sorter analysis of the percentage of muscle cells expressing tdT (n = 5). Right panel: Quantifications of the percentage of tdT$^+$ (Gli1$^+$) cells (n = 5). **c** Left panel: Fluorescence-activated cell sorter analysis of the percentage of MuSCs expressing tdT (n = 5). Right panel: Quantifications of the percentage of tdT$^+$ (Gli1$^+$) MuSCs (n = 5). **d** Scheme of the experimental strategy. **e** Reconstructed 3D images of the EDL of Gli1-CreERT2; R26-eGFP; Pax7-DreERT2; R26-RSR-tdT mice at 14 days after TAM treatment (n = 3). Images were obtained by Leica TCS SP8 confocal microscope (z-stack: 1 μm/slice, 200 slices). White arrows indicate the GFP$^+$tdT$^+$ (Gli1$^+$) cells. Scale bar, 20 μm. **f** Fluorescence-activated cell sorter analysis of the percentage of muscle cells expressing tdT and the percentage of tdT$^+$ (Pax7$^+$) cells expressing GFP(Gli1) (n = 3). **g** Quantification of the mean myofiber cross-section area (CSA μm^2, n = 3). **h** Left panel: Immunofluorescence staining for tdT (red), GFP (green), Pax7 (gray) and DAPI (blue) in TA muscle sections from NOD-SCID mice at day 30 after transplantation (n = 5). Scale bars, 50 μm. Right panel: Quantification of the percentage of Pax7$^+$Gli1$^+$ and Pax7$^+$Gli1$^-$ cells at 30 days after transplantation (n = 5). Data are presented as mean ± SEM; Statistical significance was determined by two-tailed unpaired Student's *t* test (**b, c, h**) or one-way ANOVA (**d**). All numbers (n) are biologically independent experiments. Source data is provided in the Source Data File.

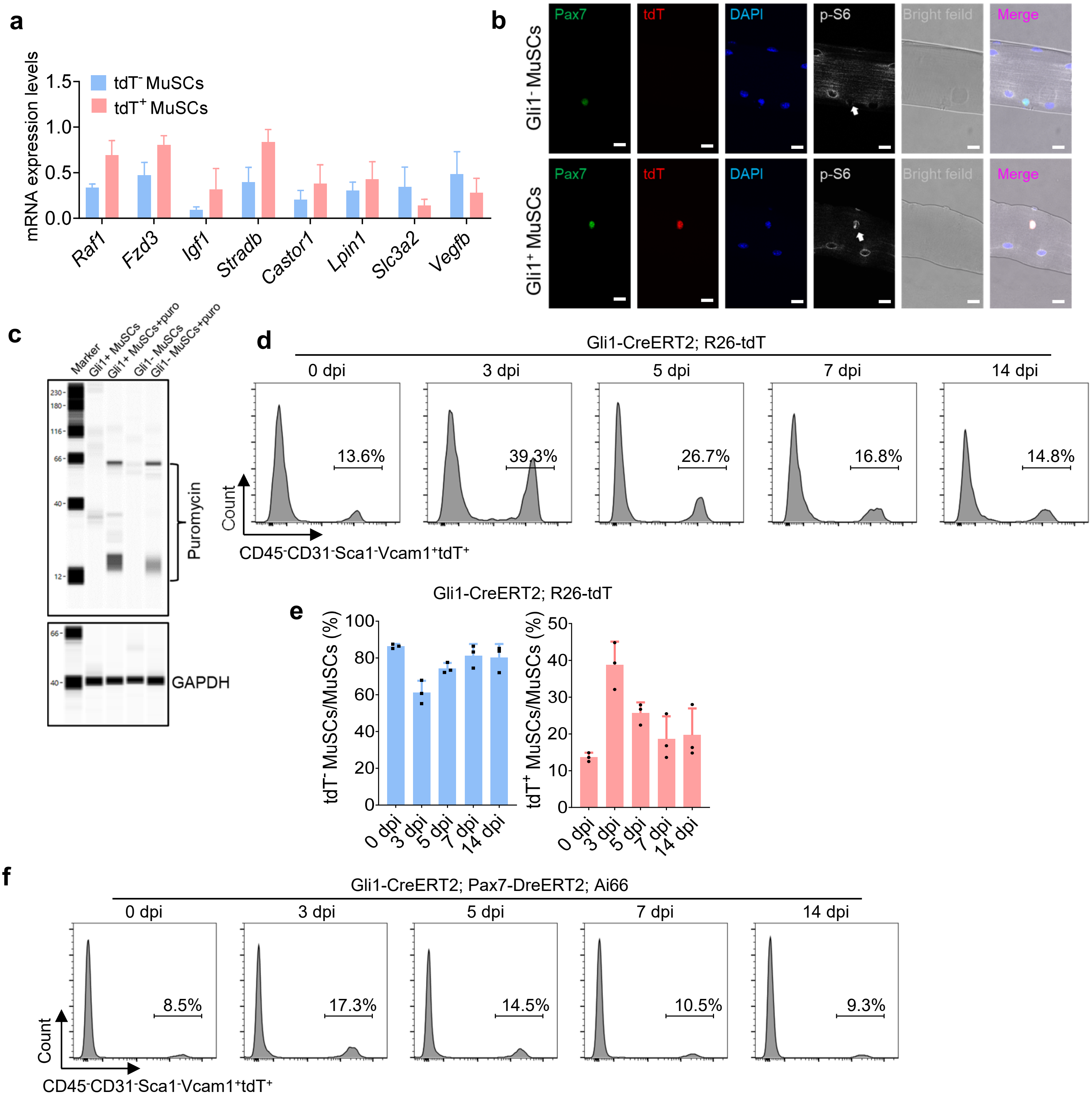

a
mRNA expression levels
tdT- MuSCs
tdT+ MuSCs
Raf1
Fzd3
Igf1
Stradb
Castor1
Lpin1
Slc3a2
Vegfb
b
Gli1- MuSCs
Gli1+ MuSCs
Pax7
tdT
DAPI
p-S6
Bright feild
Merge
c
Marker
Gli1+ MuSCs
Gli1+ MuSCs+puro
Gli1- MuSCs
Gli1- MuSCs+puro
Puromycin
GAPDH
d
Gli1-CreERT2; R26-tdT
0 dpi
3 dpi
5 dpi
7 dpi
14 dpi
13.6%
39.3%
26.7%
16.8%
14.8%
Count
CD45-CD31-Sca1-Vcam1+tdT+
e
Gli1-CreERT2; R26-tdT
tdT- MuSCs/MuSCs (%)
tdT+ MuSCs/MuSCs (%)
f
Gli1-CreERT2; Pax7-DreERT2; Ai66
0 dpi
3 dpi
5 dpi
7 dpi
14 dpi
8.5%
17.3%
14.5%
10.5%
9.3%
Count
CD45-CD31-Sca1-Vcam1+tdT+

Supplementary Fig. 9 $Gli1^+$ MuSCs display elevated mTOR signaling.

a Relative mRNA levels of the mTOR signal pathway genes were measured by RT-qPCR (n = 3). The red histogram represents gene expression level in tdT^+ ($Gli1^+$) MuSCs. The blue histogram represents gene expression level in tdT^- ($Gli1^-$) MuSCs. **b** Immunofluorescence staining for tdT (red), Pax7 (green), p-S6 (gray) and DAPI (blue) on the freshly isolated single EDL myofibers from Gli1-CreERT2; R26-tdT mice (n = 5). White arrows indicate the tdT^+pS6^+ cells. Scale bars, 10 μm. **c** Western blot for Puromycin and GAPDH (loading control) from tdT^+ ($Gli1^+$) and tdT^- ($Gli1^-$) MuSCs using a capillary-based western blot automated system. **d** FACS analysis of the percentage of $CD45^-CD31^-Sca1^-Vcam1^+tdT^+$ MuSCs in total MuSCs from Gli1-CreERT2; R26-tdT mice at different time points after CTX injury (n = 3). dpi, days post-injury. **e** Quantification of the percentage of tdT^+ ($Gli1^+$) and tdT^- ($Gli1^-$) MuSCs from Gli1-CreERT2; R26-tdT mice at different time points after CTX injury. The red histogram represents the percentage of tdT^+ ($Gli1^+$) MuSCs (n = 3). The blue histogram represents the percentage of tdT^- ($Gli1^-$) MuSCs. dpi, days post-injury. **f** FACS analysis of the percentage of $CD45^-CD31^-Sca1^-Vcam1^+tdT^+$ MuSCs in total MuSCs from Gli1-CreERT2; Pax7-DreERT2; Ai66 mice at different time points after CTX injury (n = 3). dpi, days post-injury. Data are presented as mean ± SEM. All numbers (n) are biologically independent experiments. Source data is provided in the Source Data File.

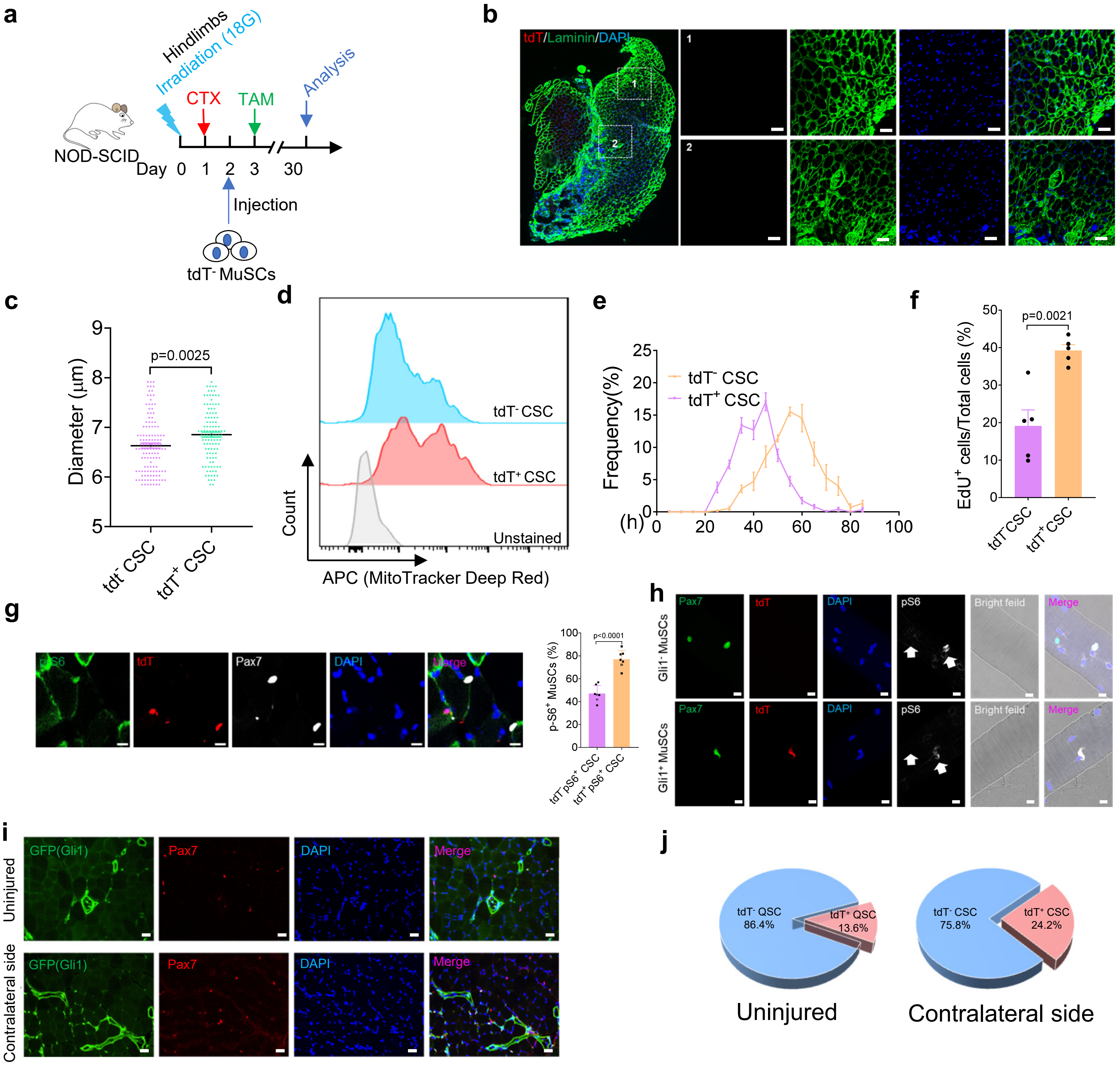
a
Hindlimbs
Irradiation (18G)
CTX
TAM
Analysis
NOD-SCID
Day 0 1 2 3 30
Injection
tdT- MuSCs
b
tdT/Laminin/DAPI
c
Diameter (μm)
p=0.0025
tdt- CSC
tdT+ CSC
d
tdT- CSC
tdT+ CSC
Unstained
Count
APC (MitoTracker Deep Red)
e
Frequency(%)
tdT- CSC
tdT+ CSC
(h)
f
EdU+ cells/Total cells (%)
p=0.0021
tdT-CSC
tdT+CSC
g
pS6
tdT
Pax7
DAPI
Merge
p-S6+ MuSCs (%)
p<0.0001
tdT-pS6+ CSC
tdT+pS6+ CSC
h
Gli1- MuSCs
Gli1+ MuSCs
Pax7
tdT
DAPI
pS6
Bright feild
Merge
i
Uninjured
Contralateral side
GFP(Gli1)
Pax7
DAPI
Merge
j
tdT- QSC 86.4%
tdT+ QSC 13.6%
tdT- CSC 75.8%
tdT+ CSC 24.2%
Uninjured
Contralateral side

Supplementary Fig. 10 Gli1$^+$ MuSCs are function as rapid response cells after injury.

a Scheme of the experimental strategy. **b** Particular numbers (5,000 cells) of freshly isolated tdT$^-$ (Gli1$^-$) MuSCs from Gli1-CreERT2; R26-tdT mice were transplanted into pre-injured TA muscle of the NOD-SCID mice. TA muscles were harvested 30 days after transplantation. Immunofluorescence staining for tdT (red), Laminin (green) and DAPI (blue) on TA muscle sections from NOD-SCID mice (n = 3). The boxed regions are magnified in the panels 1 and 2 with split channels. Scale bars, 50 μm. **c** The diameter of from tdT$^-$ (Gli1$^-$) and tdT$^+$ (Gli1$^+$) CSCs were measured using a Coulter Multisizer 4e. n = 1,000 cells from 3 mice per group. **d** Representative plots of tdT$^+$ (Gli1$^+$) and tdT$^-$ (Gli1$^-$) CSCs stained with MitoTracker Deep Red (MTDR) (n = 5). The blue represents the tdT$^-$ (Gli1$^-$) CSCs stained with MTDR. The red represents the tdT$^+$ (Gli1$^+$) CSCs stained with MTDR. The gray represents unstained. **e** Time to the first division in tdT$^+$ (Gli1$^+$) and tdT$^-$ (Gli1$^-$) CSCs was measured by time-lapse microscopy. n = 60 cells from 3 mice per group. **f** Equal numbers of FACS-isolated tdT$^+$ (Gli1$^+$) and tdT$^-$ (Gli1$^-$) CSCs were cultured and labeled with EdU for 40 h, then immunofluorescence stained for tdT and EdU (n = 5). Quantification of the percentage of tdT$^+$ (Gli1$^+$) and tdT$^-$ (Gli1$^-$) CSCs with incorporated EdU (n = 5). **g** Left panel: Immunofluorescence staining for p-S6 (green), tdT (red), Pax7 (white) and DAPI (blue) on TA muscle sections (CSC) (n = 7). Scale bars, 10 μm. Right panel: Quantification of the percentage of tdT$^+$ (Gli1$^+$) and tdT$^-$ (Gli1$^-$) CSCs expressing p-S6 (n = 7). **h** Immunofluorescence staining for tdT (red), Pax7 (green), p-S6 (gray) and DAPI (blue) on the cultured for 24 h single EDL myofibers (n = 5). White arrows indicate the tdT$^+$pS6$^+$ or tdT$^-$pS6$^+$ cells. Scale bars, 10 μm. **i** Immunofluorescence staining for Pax7 (red), GFP (green) and DAPI (blue) on TA muscle sections of uninjured or 1 day after

CTX injury (n = 8). Scale bars, 20 μm. **j** Left panel: Quantification of the percentage of tdT$^+$ (Gli1$^+$) and tdT$^-$ (Gli1$^-$) QSCs from Gli1-CreERT2; R26-tdT mice without injury. The blue histogram represented the percentage of tdT$^-$ (Gli1$^-$) QSCs. The red histogram represented the percentage of tdT$^+$ (Gli1$^+$) QSCs. Right panel: Quantification of the percentage of tdT$^+$ (Gli1$^+$) and tdT$^-$ (Gli1$^-$) CSCs from the contralateral TA muscle of injured Gli1-CreERT2; R26-tdT mice 1 day after CTX injury. The blue histogram represented the percentage of tdT$^-$ (Gli1$^-$) CSCs. The red histogram represented the percentage of tdT$^+$ (Gli1$^+$) CSCs. Data are presented as mean ± SEM; Statistical significance was determined by two-tailed unpaired Student's *t* test (**c, f**). All numbers (n) are biologically independent experiments. Source data is provided in the Source Data File.

Supplementary Table 1: The absolute number of Gli1^{-} and Gli1^{+} MuSCs from Gli1-CreERT2; R26-tdT mice at different time points after CTX injury.

	Number (via FACS)				The absolute number		
Sample name	Beads	MuSCs	Gli1^{+} MuSCs	Gli1^{-} MuSCs	MuSCs	Gli1^{+} MuSCs	Gli1^{-} MuSCs
mouse #1-D0	23621	525	79	446	822	124	699
mouse #2-D3	166	4447	1300	3144	991199	289759	700771
mouse #3-D5	318	4233	1183	3045	492519	137645	354292
mouse #4-D7	518	3341	770	2571	238643	55000	183643
mouse #5-D14	7218	1978	304	1670	10139	1558	8561

Sample name	The number of MuSCs/All event (%)	Gli1^{+} MuSCs/All MuSCs (%)	Gli1^{-} MuSCs/All MuSCs (%)
mouse #1-D0	2.64	15	85
mouse #2-D3	61.1	29.2	70.7
mouse #3-D5	44.7	27.9	71.9
mouse #4-D7	26	23	77
mouse #5-D14	5.93	15.4	84.4

Supplementary Table 2: List of primary antibodies used for experiments

Primary antibody	Species	Dilution	Applications	Source	Catalog number
Laminin	rabbit	1:250	IF	Abcam	ab11575
Laminin	rat	1:100	IF	Abcam	ab11576
Pax7	mouse	1:100	IF	DSHB	AB_528428
PDGFRα	goat	1:100	IF	R&D	AF1062
DsRed	rabbit	1:500	IF	Clontech	632496
mcherry	goat	1:500	IF	SICGEN	AB0081-200
β-gal	rabbit	1:250	IF	Abcam	ab221199
MyHC	rabbit	1:500	IF, WB	Sigma	05-716
MyoD	mouse	1:100	IF, WB	Santa cruz	Sc-377460
MyoG	mouse	1:100	IF, WB	DSHB	AB_2146602
Myf5	rabbit	1:100	IF	Sigma	SAB4501943
eMHC	mouse	1:20	IF	DSHB	AB_528358
CD34	rabbit	1:200	IF	Abcam	ab81289
GFP (FITC-conjugated)	goat	1:400	IF	Abcam	ab6662
Gli1	rabbit	1:200	IF	NOVUS	NB600-600
Gli1	rabbit	1:1000	WB	CST	2534S
p-S6	rabbit	1:200	IF, WB	CST	4858S
tubulin	rabbit	1:1000	WB	Abclonal	AC015
GAPDH	rabbit	1:1000	WB	CST	2118S
CD45 (FITC-conjugated)	rat	1:100	FC	BioLegend	157214
CD31 (FITC-conjugated)	rat	1:100	FC	BioLegend	160212
Sca1 (FITC-conjugated)	rat	1:100	FC	BioLegend	108106
VCAM1 (APC-conjugated)	rat	1:100	FC	BioLegend	105717
VCAM1 (PE/cy7-conjugated)	rat	1:100	FC	BioLegend	105720

Supplementary Table 3: List of primer sequences used for RT-qPCR

Gene	Pimer sequences (5'-3')
Raf1	Fw - CTCTGAAGGTGAGAGGCCTG<br>Rv - CGGCATCGGTGTTCCAATCT
Fzd3	Fw - GTTGCAGTGCAGAGGGACTA<br>Rv - GGACATGGTGGCGAACAATC
Igf1	Fw - CTGGACCAGAGACCCTTTGC<br>Rv - GGACGGGGACTTCTGAGTCTT
Stradb	Fw - CTTGACCTCTGTTCATCTTGCAC<br>Rv - GGAGAAATAACCCAAAGCCAGC
Castor1	Fw - GAGTACTGAGCATTGCCCGT<br>Rv - CAGGCTGAAGAACTTGCACC
Lpin1	Fw - CTCCGCTCCCGAGAGAAAG<br>Rv - TCATGTGCAAATCCACGGACT
Slc3a2	Fw - GACACCGAAGTGGACATGAAA<br>Rv - GCTCCTCCTTGGATAAGCCG
Stk11	Fw - TGGGCATGGACACCTTCATC<br>Rv - AGGTCCCCCATCAGGTACTT
Vegfb	Fw - GCCAGACAGGGTTGCCATAC<br>Rv - GGAGTGGGATGGATGATGTCAG
Pax7	Fw - TCTCCAAGATTCTGTGCCGAT<br>Rv - CGGGGTTCTCTCTCTTATACTCC
MyoD	Fw - ATGATGACCCGTGTTTCGACT<br>Rv - CACCGCAGTAGGGAAGTGT
MyoG	Fw - GCAGGCTCAAGAAAGTGAATGA<br>Rv - TAGGCGCTCAATGTACTGGAT

Myf5	Fw - GCCTTCGGAGCACACAAAG<br>Rv - TGACCTTCTTCAGGCGTCTAC
Cd34	Fw - AAGGCTGGGTGAAGACCCTTA<br>Rv - TGAATGGCCGTTTCTGGAAGT
Hhip	Fw - TGAAGATGCTCTCGTTTAAGCTG<br>Rv - CCACCACACAGGATCTCTCC
Gli3	Fw - GAAGAAACGCAATCACTATGCAG<br>Rv - GTCCCACGGTAAGGGAGAGA
Ptch1	Fw - AAAGAACTGCGGCAAGTTTTTG<br>Rv - CTTCTCCTATCTTCTGACGGGT
Gli1	Fw - CCAAGCCAACTTTATGTCAGGG<br>Rv - AGCCCGCTTCTTTGTTAATTTGA
Gapdh	Fw - AGGTCGGTGTGAACGGATTTG<br>Rv - TGTAGACCATGTAGTTGAGGTCA
