# Supplementary material for: Gli1 marks a sentinel muscle stem cell population for muscle regeneration
Source: Nat Commun. 2023 Nov 1;14:6993. doi: 10.1038/s41467-023-42837-8 (PMC10620419; doi:10.1038/s41467-023-42837-8)
Supplement: Supplementary file 3 — Reporting Summary [file 41467_2023_42837_MOESM3_ESM.pdf]

nature portfolio

Corresponding author(s): Yun Zhao, Ping Hu

Last updated by author(s): Oct 6, 2023

Reporting Summary

Nature Portfolio wishes to improve the reproducibility of the work that we publish. This form provides structure for consistency and transparency in reporting. For further information on Nature Portfolio policies, see our Editorial Policies and the Editorial Policy Checklist.

Statistics

For all statistical analyses, confirm that the following items are present in the figure legend, table legend, main text, or Methods section.

n/a	Confirmed	
☐	☒	The exact sample size (*n*) for each experimental group/condition, given as a discrete number and unit of measurement
☐	☒	A statement on whether measurements were taken from distinct samples or whether the same sample was measured repeatedly
☐	☒	The statistical test(s) used AND whether they are one- or two-sided<br>*Only common tests should be described solely by name; describe more complex techniques in the Methods section.*
☒	☐	A description of all covariates tested
☒	☐	A description of any assumptions or corrections, such as tests of normality and adjustment for multiple comparisons
☐	☒	A full description of the statistical parameters including central tendency (e.g. means) or other basic estimates (e.g. regression coefficient) AND variation (e.g. standard deviation) or associated estimates of uncertainty (e.g. confidence intervals)
☐	☒	For null hypothesis testing, the test statistic (e.g. *F*, *t*, *r*) with confidence intervals, effect sizes, degrees of freedom and *P* value noted<br>*Give P values as exact values whenever suitable.*
☒	☐	For Bayesian analysis, information on the choice of priors and Markov chain Monte Carlo settings
☒	☐	For hierarchical and complex designs, identification of the appropriate level for tests and full reporting of outcomes
☒	☐	Estimates of effect sizes (e.g. Cohen's *d*, Pearson's *r*), indicating how they were calculated

Our web collection on statistics for biologists contains articles on many of the points above.

Software and code

Policy information about availability of computer code

Data collection	Olympus fluorescence microscope (BX53) and Leica TCS SP8 confocal microscope were used for immunofluorescence data collection. FACS Aria III (BD Biosciences) was used for FACS data collection. WB were examined using automated western blot system, WES System (ProteinSimple). The cell size of MuSCs were measured by Coulter Multisizer 4e (Beckman). Time to the first division was measured by time-lapse microscopy (ZEISS Celldiscoverer 7). RNA-seq and Single cell RNA-seq libraries were constructed according to the manufacturers' instructions, and the constructed libraries were sequenced on Illumina Nextseq 500 sequencer.
Data analysis	Image J(FIJI) was used for immunofluorescence and bright-filed images analysis. FlowJo_V10 was used for flow cytometer data analysis. GraphPad Prism 8.0 was used for data analysis. Data analysis and quantitation of protein levels were performed using Compass Software (ProteinSimple). Cell Ranger (v.2.1.1) were used for demultiplexing , barcode processing read alignment , gene counting and generation of gege-barcode expression matrix. Seurat (v.3.1.1) was used to analyze single cell RNA-seq data. RNA-seq reads were mapped to transcripts from Mus_musculus.GRCm38.84 by STAR (star-2.7). Differential expression was assessed using DESeq2 package. To further compare biological function of MuSCs, Metascape (http://metascape.org/gp/index.html) were performed.

For manuscripts utilizing custom algorithms or software that are central to the research but not yet described in published literature, software must be made available to editors and reviewers. We strongly encourage code deposition in a community repository (e.g. GitHub). See the Nature Portfolio guidelines for submitting code & software for further information.

Data

Policy information about availability of data

All manuscripts must include a data availability statement. This statement should provide the following information, where applicable:

- Accession codes, unique identifiers, or web links for publicly available datasets
- A description of any restrictions on data availability
- For clinical datasets or third party data, please ensure that the statement adheres to our policy

The Single cell RNA-sequencing raw data generated in this study have been deposited in the National Center for Biotechnology Information (NCBI) Sequence Read Archive (SRA) database under BioProject accession number PRJNA1028159 (https://www.ncbi.nlm.nih.gov/sra/PRJNA1028159). The RNA sequencing raw data generated in this study have been deposited in the National Center for Biotechnology Information (NCBI) database under accession code GSE239944 (https://www.ncbi.nlm.nih.gov/geo/query/acc.cgi?acc=GSE239944). The GRCm38 data used in this study are available in the NCBI database under accession code GCF_000001635.20. Source data are provided with this paper.

Research involving human participants, their data, or biological material

Policy information about studies with human participants or human data. See also policy information about sex, gender (identity/presentation), and sexual orientation and race, ethnicity and racism.

Reporting on sex and gender	n/a
Reporting on race, ethnicity, or other socially relevant groupings	n/a
Population characteristics	n/a
Recruitment	n/a
Ethics oversight	n/a

Note that full information on the approval of the study protocol must also be provided in the manuscript.

Field-specific reporting

Please select the one below that is the best fit for your research. If you are not sure, read the appropriate sections before making your selection.

☒ Life sciences ☐ Behavioural & social sciences ☐ Ecological, evolutionary & environmental sciences

For a reference copy of the document with all sections, see nature.com/documents/nr-reporting-summary-flat.pdf

Life sciences study design

All studies must disclose on these points even when the disclosure is negative.

Sample size	For all experiments, n ≥ 3 was used according to standard scientific conventions, each sample size were described in detail in each figure legend. No statistical methods were used to predetermine the sample size.
Data exclusions	No data were excluded from the analysis
Replication	All in vivo and in vitro experiments were highly reproducible and were independently repeated at least 3 times. All experiments were performed independently multiple times using biologically independent replicates. All attempts at replication were successful.
Randomization	Mice were randomly assigned to groups. Cells were grown under the same conditions and randomly allocated into different groups without any bias.
Blinding	The investigators were blinded to group allocation during data collection and analysis. We collected and analyzed the compared samples under the same conditions.

Reporting for specific materials, systems and methods

We require information from authors about some types of materials, experimental systems and methods used in many studies. Here, indicate whether each material, system or method listed is relevant to your study. If you are not sure if a list item applies to your research, read the appropriate section before selecting a response.

Materials & experimental systems

n/a	Involved in the study
☐	☒ Antibodies
☒	☐ Eukaryotic cell lines
☒	☐ Palaeontology and archaeology
☐	☒ Animals and other organisms
☒	☐ Clinical data
☒	☐ Dual use research of concern
☒	☐ Plants

Methods

n/a	Involved in the study
☒	☐ ChIP-seq
☐	☒ Flow cytometry
☒	☐ MRI-based neuroimaging

Antibodies

Antibodies used

1. Primary antibodies used in immunofluorescence for this study included:
Laminin (rabbit 1:250 Abcam ab11575), Laminin (rat 1:100 Abcam ab11576), Pax7 (mouse 1:100 DSHB AB_528428), PDGFRα (goat 1:100 R&D AF1062), DsRed (rabbit 1:500 Clontech 632496), mcherry (goat 1:500 SICGEN AB0081-200), β-gal (rabbit 1:250 Abcam ab221199), MyHC (rabbit 1:500 Sigma 05-716), p-S6 (rabbit 1:200 CST 4858S), MyoD (mouse 1:100 Santa cruz Sc-377460), MyoG (mouse 1:100 DSHB AB_2146602), Myf5 (rabbit 1:100 Sigma SAB4501943), eMHC (mouse 1:20 DSHB AB_528358), CD34 (rabbit 1:200 Abcam ab81289), GFP (FITC-conjugated) (goat 1:400 Abcam ab6662), Gli1 (rabbit 1:200 NOVUS NB600-600).
2. Primary antibodies used in WB for this study included:
p-S6 (rabbit 1:500 CST 4858S), tubulin (rabbit 1:1000 Abclonal AC015), MyHC (rabbit 1:500 Sigma 05-716), Gli1 (rabbit 1:1000 CST 2534S), MyoD (mouse 1:1000 Santa cruz Sc-377460), MyoG (mouse 1:1000 DSHB AB_2146602), GAPDH (rabbit 1:1000 CST 2118S).
3. Primary antibodies used in FACS for this study included:
CD45-FITC (rat 1:100 BioLegend 157214), CD31--FITC (rat 1:100 BioLegend 160212), Sca1-FITC (rat 1:100 BioLegend 108106),VCAM1-APC (rat 1:100 BioLegend 105717), VCAM1-PE/cy7 (rat 1:100 BioLegend 105720).
4.The included secondary antibodies were Alexa donkey anti-mouse 555 (Invitrogen A31570), Alexa donkey anti-rabbit 488 (Invitrogen A21206), Alexa donkey anti-goat 555 (Invitrogen A21432), Alexa donkey anti-goat 647 (Invitrogen A21447), Alexa donkey anti-rabbit 555 (Invitrogen A31572), Alexa donkey anti-rat 555 (Invitrogen A21434). Opal 3-Plex Manual Detection Kit (Akoya NEL810001KT).

Validation

Antibodies were all sourced commercially with independent validations and citations.

Animals and other research organisms

Policy information about studies involving animals; ARRIVE guidelines recommended for reporting animal research, and Sex and Gender in Research

Laboratory animals

Mice were aged 8-12 weeks at the start of experiments.
All mice used for experiments were kept at C57BL6/129 mixed background.
The strains included Gli1-CreERT2;R26-tdT, Pax7-DreERT2;R26-RSR-tdT, Gli1-CreERT2;Ai66, Pax7-DreERT2;Ai66, Gli1-CreERT2;Pax7-DreERT2;Ai66, Gli1-CreERT2;R26-eGFP, Gli1-CreERT2;R26-eGFP;Pax7-DreERT2;R26-RSR-tdT, Gli1-CreERT2;R26-DTA, DMD and NOD-Scid.
All mice were kept in group housing (2-5 mice per cage) in a specific pathogen-free (SPF) facility with controlled environmental conditions of temperature (20-25°C), humidity (40-70%) and lighting (a 12h light/dark cycle) at Center for Excellence in Molecular Cell Science, University of Chinese Academy of Sciences, Chinese Academy of Sciences.

Wild animals

The study did not involve wild animals.

Reporting on sex

Both male and female mice were used. Sex was not considered in this study design.

Field-collected samples

The study did not involve samples collected from the field.

Ethics oversight

Mice were housed and maintained in accordance with the guidelines of the Institutional Animal Care and Use Committee of the State Key Laboratory of Cell Biology, Shanghai Institute of Biochemistry and Cell Biology, Center for Excellence in Molecular Cell Science, Chinese Academy of Sciences.

Note that full information on the approval of the study protocol must also be provided in the manuscript.

Flow Cytometry

Plots

Confirm that:

☒ The axis labels state the marker and fluorochrome used (e.g. CD4-FITC).

☒ The axis scales are clearly visible. Include numbers along axes only for bottom left plot of group (a 'group' is an analysis of identical markers).

☒ All plots are contour plots with outliers or pseudocolor plots.

☒ A numerical value for number of cells or percentage (with statistics) is provided.

Methodology

Sample preparation	For isolation of MuSCs by FACS, after mice were euthanized, the hindlimb skeletal muscles were removed, chopped finely, and digested using 700 U/ml Collagenase type 2 (Worthington) in Wash medium (Ham's F10 supplemented with 1% Penicillin-Streptomycin and 10% horse serum) for 60 min in shaking water bath at 37°C. The digested muscle was washed twice with Wash medium and centrifuged at 500 g at 4°C for 5 min. A second digestion was performed with 1000 U/ml Collagenase type II and 1.1 U/ml Dispase II in Wash medium for 30 min in shaking water bath at 37°C. The twice-digested tissue was passed through a 20-gauge needle three times, then passed through a 70 µm filter and a 40 µm filter. The mononuclear muscle cells were stained using the following antibodies: FITC anti-mouse CD31, FITC anti-mouse CD45, FITC anti-mouse Sca1, APC or PE/cy7 anti-mouse CD106/VCAM1. Cells were incubated with primary antibodies for 60 min on ice, washed with cold Wash medium. DAPI were used for viable cell gating. FACS was performed using FACS Aria III (BD Biosciences) by gating for CD45-CD31-Sca1-VCAM1+ to isolate MuSCs. Gli1+ or Gli1- MuSCs were sorted based on CD45-CD31-Sca1-VCAM1+tdT+ or CD45-CD31-Sca1-VCAM1+tdT-.
Instrument	Data were collected by FACS Aria III (BD Biosciences).
Software	The raw data were processed by FlowJo_V10 software.
Cell population abundance	FACS-isolated populations had a purity of >97%.
Gating strategy	FSC vs SSC was used to exclude cellular debris and doublets. MuSCs were isolated by sorting for viability stain (DAPI-) and lineage (CD45-CD31-Sca1-Vcam1+) cells. Unstained controls were used to draw the lineage-negative gate.

☒ Tick this box to confirm that a figure exemplifying the gating strategy is provided in the Supplementary Information.
