# Supplementary figures and images for: Gli1 marks a sentinel muscle stem cell population for muscle regeneration
Source: Nat Commun. 2023 Nov 1;14:6993. doi: 10.1038/s41467-023-42837-8 (PMC10620419; doi:10.1038/s41467-023-42837-8)

**Figure 3d**

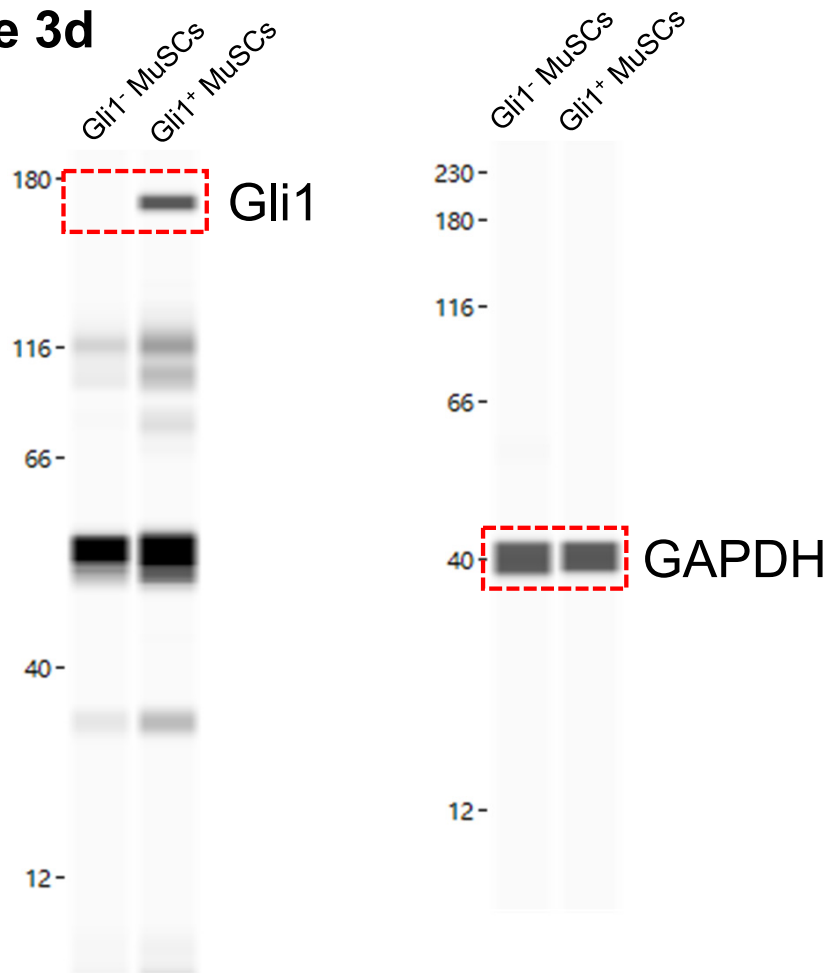

**Figure 3j**

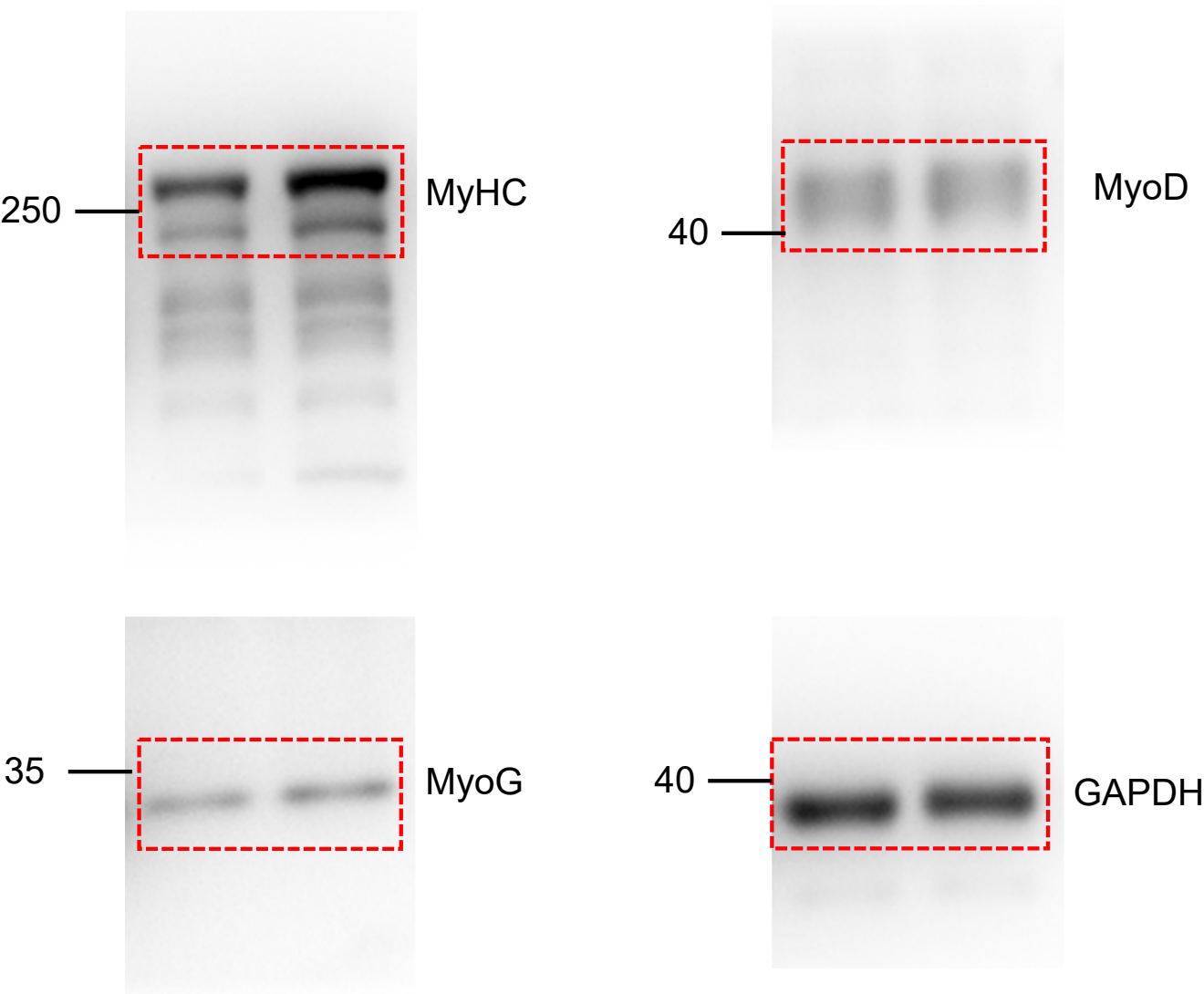

Figure 7c

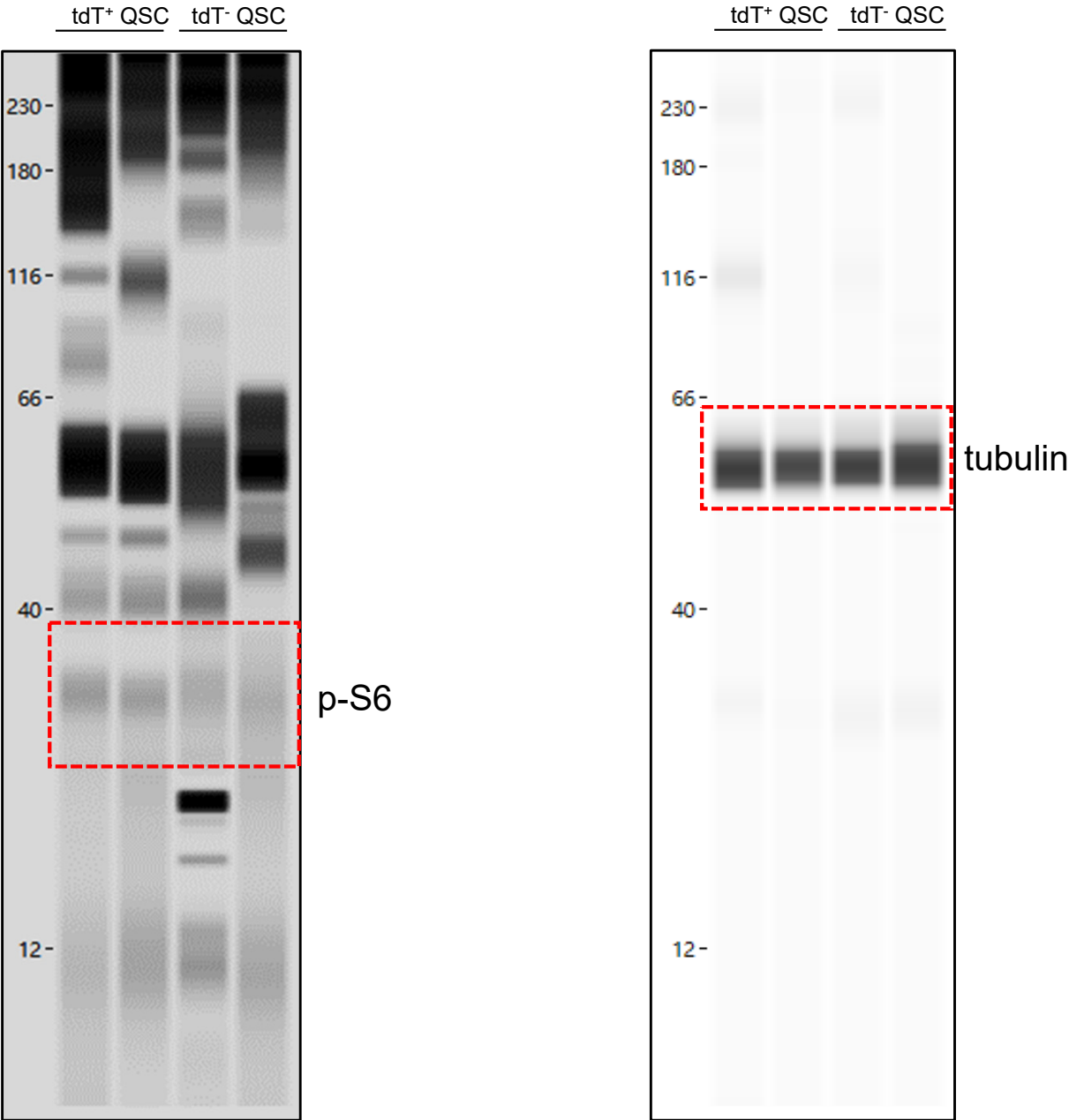

Figure S9c

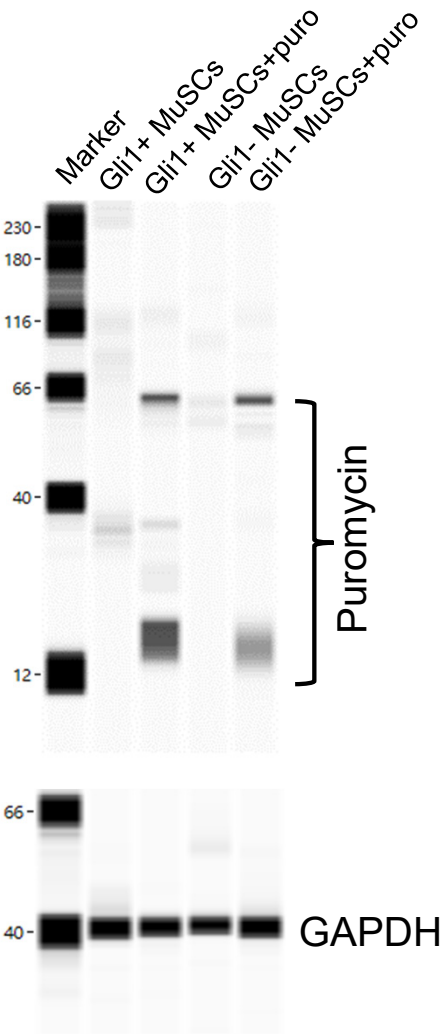

Supplement: Supplementary file 4 — Source Data [file 41467_2023_42837_MOESM4_ESM.zip › Source Data/blots.pdf]
